# Synergistic Value of Combined Electrical, Structural, and Functional Response Metrics in the Prediction of Long-Term Event-Free Survival After Cardiac Resynchronization Therapy

**DOI:** 10.31083/RCM52157

**Published:** 2026-07-20

**Authors:** Amr Yosry Emam, Ahmad Samir, Karimeldeen Hafez, Mahmoud Abdelfattah, Rehab Mohamed Elnagar, Shaimaa Nabil, Amany Ellithy, Ekram Abdelmaged Abdelhafez, Doha Ashraf Eltanahy, Eslam Ahmed, Omnia Kamel

**Affiliations:** ^1^Department of Adult Cardiology, Aswan Heart Centre, 1242770 Aswan, Egypt; ^2^Cardiology Department, Faculty of Medicine, Cairo University, 12613 Cairo, Egypt; ^3^Department of Radiology and Advanced Cardiac Imaging, Aswan Heart Center, 1242770 Aswan, Egypt; ^4^Department of Radiodiagnosis and Medical Imaging, Faculty of Medicine, Tanta University, 6632120 Tanta, Egypt; ^5^Nursing Department, Aswan Heart Centre, 1242770 Aswan, Egypt; ^6^Quality Improvement and Risk Management Department, Aswan Heart Centre, 1242770 Aswan, Egypt

**Keywords:** cardiac resynchronization therapy, heart failure, reverse remodeling, mortality

## Abstract

**Background:**

Cardiac resynchronization therapy (CRT) is a cornerstone in the management of patients with heart failure with reduced ejection fraction (HFrEF) and electrical dyssynchrony. This study aimed to identify clinical, electrocardiographic (ECG), biomarker, echocardiographic, and cardiac magnetic resonance (CMR) imaging parameters predictive of 2-year all-cause mortality and hospitalization following CRT implantation.

**Methods:**

We retrospectively analyzed data from patients undergoing CRT. Baseline characteristics and 6–12-month follow-up data, including biomarkers, ECG parameters, echocardiographic measures, and CMR parameters, were assessed. Univariate and multivariate regression analyses were performed to identify predictors of 2-year event-free survival, defined as freedom from all-cause mortality or hospitalization.

**Results:**

At baseline, higher N-terminal pro-B-type natriuretic peptide (NT-proBNP) and creatinine levels, impaired right ventricular ejection fraction (RVEF), increased right ventricular end-systolic volume (RVESV), and reduced left ventricular global longitudinal strain (LV GLS) were significantly associated with adverse outcomes. At follow-up, event-free patients had lower NT-proBNP levels, higher left ventricular ejection fraction (LVEF), and lower left ventricular end-systolic volume (LVESV). Response rates for QRS complex (QRS) shortening (≥20 ms), LVEF improvement (≥10% absolute increase), and LVESV reduction (≥15%) were significantly higher in the event-free group. Univariate predictors of 2-year event-free survival included baseline RVEF (odds ratio (OR) 0.958; *p* = 0.015), baseline LV GLS (OR 1.256; *p* = 0.047), and follow-up LVEF (OR 0.932; *p* = 0.006). In contrast, higher follow-up Right Ventricular End-systolic Volume Indexed (RVESVi) (OR 1.013; *p* = 0.048) was associated with increased risk. A multivariate model incorporating QRS duration shortening (≥20 ms), LVESV reduction (≥15%), and LVEF improvement (≥10%) was highly predictive of 2-year event-free survival (*p* = 0.006; area under the curve (AUC) 0.835, accuracy 84%, specificity 97.5%, sensitivity 30%), with a QRS duration response ≥20 ms emerging as the strongest independent predictor (adjusted OR 0.111; *p* = 0.02).

**Conclusion:**

Combined improvement in QRS duration, LVESV, and LVEF after CRT implantation is a strong predictor of 2-year event-free survival. These findings support a comprehensive, multiparameter approach to evaluating CRT efficacy and guiding clinical management.

## 1. Introduction

Cardiac resynchronization therapy (CRT) is a cornerstone treatment for patients with heart failure with reduced ejection fraction (HFrEF), and electrical dyssynchrony, demonstrating significant reductions in morbidity and mortality in many pivotal trials [1,2,3]. Evaluation of these studies shows that as many as 30–40% of patients do not respond to or even deteriorate after CRT despite tight selection criteria [4]. Traditional predictors of CRT success, such as QRS narrowing, left ventricular ejection fraction (LVEF) improvement, or biomarker reduction, are often evaluated in isolation [5,6,7]. While these individual metrics reflect facets of CRT response—electrical resynchronization, functional improvement, and reverse remodeling, respectively—their standalone predictive value for long-term clinical outcomes remains inconsistent and insufficiently robust [7,8]. Emerging evidence suggests that CRT efficacy manifests through multidimensional physiological improvements [9], yet a standardized, integrative approach to quantify this synergy is lacking. We hypothesized that a composite assessment of electrical resynchronization (i.e., QRS narrowing), LV reverse remodeling (i.e., left ventricular end-systolic volume (LVESV) reduction), and functional improvement (i.e., LVEF improvement) would outperform any single parameter in predicting 2-year event-free survival.

## 2. Methods

This retrospective cohort study included 96 consecutive patients with HFrEF who underwent CRT implantation between January 2015 and July 2023 and had a cardiac magnetic resonance (CMR) study within 60 days prior to implantation. CMR data were unavailable in a subset of patients due to device incompatibility, renal dysfunction, or logistical limitations, which may introduce selection bias. Inclusion criteria were: (i) CRT eligibility was based on guideline-directed criteria: LVEF ≤35%, QRS prolongation (≥130 ms with left bundle branch block (LBBB) or ≥150 ms with non-LBBB), and persistent symptoms despite optimal medical therapy, (ii) CRT for the indication of HFrEF management. Exclusion criteria were: (1) CRT for pacing indications, (2) Major non-cardiac comorbidity [e.g., metastatic cancer] clearly leading to an estimated life expectancy of <2-years. The study complied with the Declaration of Helsinki and received approval from the institutional ethics review board (REC Approval number: 20241031MYFAHC_CRTFP20250213). Given the retrospective design, and the observational analytic nature of the study, the institutional ethics committee approved waiving of specialized patients' consent for study participation. Patients were eligible if they were ≥18 years old with guideline-directed CRT indications (LVEF ≤35%, QRS duration ≥130 ms, New York Heart Association (NYHA) Class II–IV on optimal medical therapy). Exclusion criteria included active infection, pregnancy, or non-cardiac life expectancy <2 years.

The primary endpoint was 2-year event-free survival, defined as freedom from all-cause mortality or all-cause hospitalization. Participants were stratified into two groups: an event-free group (n = 75) with no endpoint events at 2 years, and an event group (n = 21) experiencing endpoint events within 2 years.

Baseline assessments conducted pre-implantation included collection of clinical/demographic variables, functional capacity evaluation via 6-minute walk test, biomarker analysis (NT-proBNP, creatinine, estimated glomerular filtration rate (eGFR)), 12-lead electrocardiographic (ECG) measurements, comprehensive echocardiography, and cardiac magnetic resonance imaging (MRI) with strain analysis. Follow-up evaluations at 6–12 months post-implantation repeated ECG, echocardiographic measures, assessed device pacing percentages, and tracked response variables. Response criteria were predefined as: (i) QRS response: ≥20 ms narrowing, (ii) LVEF response: ≥10% absolute improvement, (iii) LVESV response: ≥15% reduction, and (iv) Brain Natriuretic Peptide (BNP) response: ≥30% NT-proBNP decline. QRS duration at baseline was measured on intrinsic rhythm ECG prior to CRT implantation. At follow-up, QRS duration was measured on paced ECG recordings, reflecting the electrical effect of biventricular pacing with Biventricular (BiV) pacing achieving ≥95% in the majority of our patients by the time of assessment. Cut off values were selected based on prior CRT literature which are commonly used thresholds to define meaningful electrical and reverse remodeling response [10]. All patients were treated with guideline-directed medical therapy at the time of CRT implantation. However, detailed longitudinal data on medication dose optimization during follow-up were not uniformly available and were therefore not included in multivariable modeling.

### Statistical Analysis

Statistical analysis employed Student’s *t*-tests or Mann-Whitney U tests for continuous variables (normality assessed by Shapiro-Wilk) and Chi-square/Fisher’s exact tests for categorical variables. Baseline variables were analyzed as predictors of prognosis, whereas follow-up parameters were evaluated as post-implant treatment response markers and interpreted accordingly.

Univariate logistic regression identified potential predictors (*p* < 0.10 threshold for multivariate entry), with backward elimination multivariate modeling (*p* < 0.05 retention). Model performance was evaluated via Receiver Operating Characteristic (ROC) curves (AUC), sensitivity, specificity, and accuracy. Analyses used SPSS v27.0 (IBM Corp., Armonk, NY, USA) and R v4.1.2 (R Foundation for Statistical Computing, Vienna, Austria), with 80% power (α = 0.05) to detect AUC ≥0.75 for composite predictors given the cohort size.

## 3. Results

### 3.1 Baseline Characteristics

A total of 96 patients undergoing CRT implantation were stratified into event-free survivors (n = 75) and those experiencing all-cause mortality or hospitalization within 2 years (n = 21). At baseline, the event group exhibited significantly higher NT-proBNP levels (2482 vs. 1109 pg/mL; *p* = 0.012), elevated creatinine (1.10 vs. 0.80 mg/dL; *p* = 0.028), higher E/A (3.0 ± 1.07 vs. 1.1 ± 0.76; *p* = 0.015) and impaired right ventricular (RV) function on cardiac MRI, reflected by lower RV ejection fraction (38.5 ± 16.4% vs. 45 ± 17.5%; *p* = 0.007) and higher RV end-systolic volume (76.5 vs. 91 mL; *p* = 0.005). LV global longitudinal strain was also worse in the event group (−4.6 ± 1.88% vs. −6.25 ± 3.12%; *p* = 0.031). No significant differences were noted in LVEF, QRS duration, or comorbidities. Baseline characteristics of patients before CRT implantation are shown in Table 1.

**Table 1.** **Baseline characteristics of patients before CRT implantation**.

Variable			Event-free group (n = 75)	Event group (n = 21)	*p*-value
Demographic and clinical variables:					
	Age, years		53.0 [43–62]	49.0 [40–55]	0.169
	Gender:				0.434
		Female sex	26 (83.9%)	5 (16.1%)	
		Male sex	49 (75.4%)	16 (24.6%)	
	Weight, kg		84 ± 19.2	81.5 ± 22.2	0.643
	Height, cm		167 ± 8.4	169 ± 8.1	0.217
	BMI, kg/m^2^		30.7 ± 6.0	28.4 ± 6.9	0.259
	Smoking status:				0.488
		Ex-smoker	17 (73.9%)	6 (26.1%)	
		Non-smoker	46 (82.1%)	10 (17.9%)	
		Current smoker	12 (70.6%)	5 (29.4%)	
	Hypertension		34 (77.3%)	10 (22.7%)	0.853
	Diabetes mellitus		29 (80.6%)	7 (19.4%)	0.802
	Chronic kidney disease		24 (88.9%)	3 (11.1%)	0.253
	6MWT, m		279 [236–360]	270 [234–385]	0.858
	NT-proBNP, pg/mL		1109 [458–2627]	2482 [1908–4763]	0.012
	Creatinine, mg/dL		0.80 [0.692–1.00]	1.10 [0.80–1.40]	0.028
	eGFR, mL/min/1.73 m^2^		98.9 [76.2–121]	80.1 [65.2–110]	0.09
ECG:					
	PR, ms		180 ± 24	185 ± 22	0.438
	QRS duration, ms		160 ± 17.5	160 ± 20.0	0.761
	QTc, ms		480 ± 31	480 ± 27	0.734
Echocardiography:					
	LVEDD, cm		7.0 ± 1.26	7.1 ± 1.56	0.865
	LVESD, cm		6.1 ± 1.22	6.2 ± 1.54	0.778
	LVESV, mL		187 [124–239]	173 [108–272]	0.849
	LVEF, %		26 ± 9.8	27.5 ± 8.0	0.852
	LA diameter, cm		4.3 ± 0.76	4.9 ± 0.72	0.231
	E/A		1.1 ± 0.76	3.0 ± 1.07	0.015
	E/E'		14.0 ± 3.7	16.5 ± 6.3	0.111
	TAPSE, mm		20.5 ± 3.5	18.0 ± 3.2	0.144
	PASP, mmHg		32.0 [25.8–45.3]	45.0 [27.0–52.0]	0.315
Cardiac MRI:					
	LVEF, %		26 ± 12	23 ± 12	0.202
	LVEDV, mL		319 [271–430]	297.5 [253–487]	0.717
	LVESV, mL		239.5 [124–272]	223 [108–272]	0.793
	LV SV, mL		76 ± 26.6	72 ± 36.6	0.15
	RVEF, %		45 ± 17.5	38.5 ± 16.4	0.007
	RVEDV, mL		147 [123–186]	155.5 [121–264]	0.154
	RVESV, mL		91 [54.5–117]	76.5 [54–194]	0.005
	RV SV, mL		65 ± 24.6	65 ± 36.6	0.06
	Cardiac output, L/min		5.0 ± 1.3	5.1 ± 1.0	0.74
	Baseline CMR Scar, %		0.0 [0–2.0]	0.0 [0–8.35]	0.388
	LV mass, g		113 [98–162]	134.5 [112–186]	0.661
	LV GLS, %		−6.25 ± 3.12	−4.6 ± 1.88	0.031
	LV GCS, %		−5.4 ± 3.34	−6.2 ± 3.80	0.511
	LV GRS, %		12.2 ± 7.7	10.8 ± 6.9	0.145
	RV GLS, %		−6.7 ± 4.9	−4.4 ± 3.6	0.28
	RV GCS, %		−3.0 ± 3.2	−3.0 ± 2.8	0.792

Values are presented as mean ± standard deviation, median (interquartile range), or n (percentage). Categorical variables were compared using the Chi square test or Fisher’s exact test, as appropriate. Event-Free Group: Free of all-cause mortality/hospitalization at 2 years. Event Group: All-cause mortality/hospitalization at 2 years. 6MWT, 6 minute walk test; NT-proBNP, N-terminal pro-B-type natriuretic peptide; BMI, body mass index; eGFR, estimated glomerular filtration rate; ECG, electrocardiographic; PR, PR interval; QRS, QRS complex; QTc, QTc interval; LVEDD, left ventricular end diastolic diameter; LVESD, left ventricular end systolic diameter; LVESV, left ventricular end-systolic volume; LVEF, left ventricular ejection fraction; LA, left atrium; TAPSE, tricuspid annular plane systolic excursion; PASP, pulmonary artery systolic pressure; LVEDV, left ventricular end-diastolic volume; LV SV, left ventricular stroke volume; RVEF, right ventricular ejection fraction; RVEDV, right ventricular end diastolic volume; RVESV, right ventricular end-systolic volume; RV SV, right ventricular stroke volume; CMR, cardiac magnetic resonance; LV GLS, left ventricular global longitudinal strain; LV GCS, left ventricle global circumferential strain; LV GRS, left ventricle global radial strain; RV GLS, right ventricle global longitudinal strain; RV GCS, right ventricle global circumferential strain.

### 3.2 Follow-Up Parameters and Response Rates

Follow-up variables were analyzed as markers of treatment response rather than baseline predictors. The timing of follow-up assessment (6–12 months post-implantation) did not differ significantly between the event-free and event groups.

At 6–12 months post-implantation, the event group demonstrated persistently elevated NT-proBNP (2152.5 vs. 640 pg/mL; *p* = 0.01), reduced LVEF (32 ± 14.9% vs. 35 ± 14.0%; *p* = 0.008), and attenuated LVESV reduction (Δend systolic volume (ESV): −17.8 ± 45.2 vs. −21.2 ± 37.3 mL; *p* = 0.029). Response rates were markedly lower in the event group: (i) LVEF response (≥10% absolute improvement): 8.6% vs. 91.4%, *p* < 0.001, (ii) LVESV response (≥15% reduction): 5.3% vs. 94.7%, *p* < 0.001, (iii) QRS response (≥20 ms narrowing): 13.8% vs. 86.2%, *p* = 0.006, (iv) BNP response (≥30% NT-proBNP decline): 11.6% vs. 88.4%, *p* = 0.024, and (v) mitral regurgitation (MR) improvement (decrease at least one degree of MR severity): 13.3% vs. 86.7%, *p* = 0.01. Differences between the two groups at 6–12 months follow-up are demonstrated in Table 2.

**Table 2.** **Between-groups differences by 6–12 months follow-up**.

Variable		Event-free group (n = 75)	Event group (n = 21)	*p*-value
Biomarkers:				
	NT-proBNP, pg/mL	640 [199–2153]	2152.5 [1231–5383]	0.01
	Percent change NT-proBNP, %	−58 [−100–0]	−48 [−100–8]	0.681
ECG:				
	PR, ms	130 ± 27.9	130 ± 29.3	0.83
	QRS duration, ms	120 ± 20.9	130 ± 18.8	0.474
	QTc, ms	468 ± 42.8	472.5 ± 44.3	0.269
Device parameters:				
	Percent of biventricular pacing, %	98 ± 2.9	99 ± 2.0	0.084
Echocardiography:				
	LVEF, %	35 ± 14.0	32 ± 14.9	0.008
	LVEDD, cm	6.7 ± 1.6	7.6 ± 1.8	0.403
	LVESD, cm	5.5 ± 1.8	6.4 ± 2.0	0.185
	LVESV, mL	147.4 [78.6–216]	208.5 [119–321]	0.185
	E/A	1.1 ± 0.91	2.8 ± 1.6	0.113
	E/e'	12.0 ± 5.6	17.5 ± 6.3	0.125
	RV ś	12.0 ± 2.9	10.9 ± 2.4	0.477
	TAPSE, mm	20.5 ± 4.9	21.0 ± 4.7	0.122
	PASP, mmHg	33 ± 16.2	32 ± 9.7	0.536
	EF difference, Δ%	3.0 ± 19.3	3.0 ± 21.2	0.385
	ESV difference, Δ mL	−21.2 ± 37.3	−17.8 ± 45.2	0.029
Response rates:				
	QRS response	50 (86.2%)	8 (13.8%)	0.006
	EF response	53 (91.4%)	5 (8.6%)	<0.001
	LVESV response	54 (94.7%)	3 (5.3%)	<0.001
	BNP response ≥30%	38 (88.4%)	5 (11.6%)	0.024
	MR improvement	52 (86.7%)	8 (13.3%)	0.010

Values are presented as mean ± standard deviation, median (interquartile range), or n (percentage). Event-Free Group: Free of all-cause mortality/hospitalization at 2 years. Event Group: All-cause mortality/hospitalization at 2 years. ΔLV ESV: Change in left ventricular end-systolic volume from baseline. Response Definitions: QRS response is defined as shortening of QRS duration ≥20 ms after CRT implantation, EF response is defined as ≥10% absolute increase in LVEF after CRT implantation, LVESV response is defined as ≥15% reduction in LVESV after CRT implantation. BNP response ≥30% NT-proBNP decline. MR improvement is defined as a decrease at least one degree of MR severity. RV ś, Right Ventricle ś; EF, ejection fraction; ESV, end systolic volume; MR, mitral regurgitation; BNP, brain natriuretic peptide.

### 3.3 Predictors of 2-Year Events

**Fig. 1.**
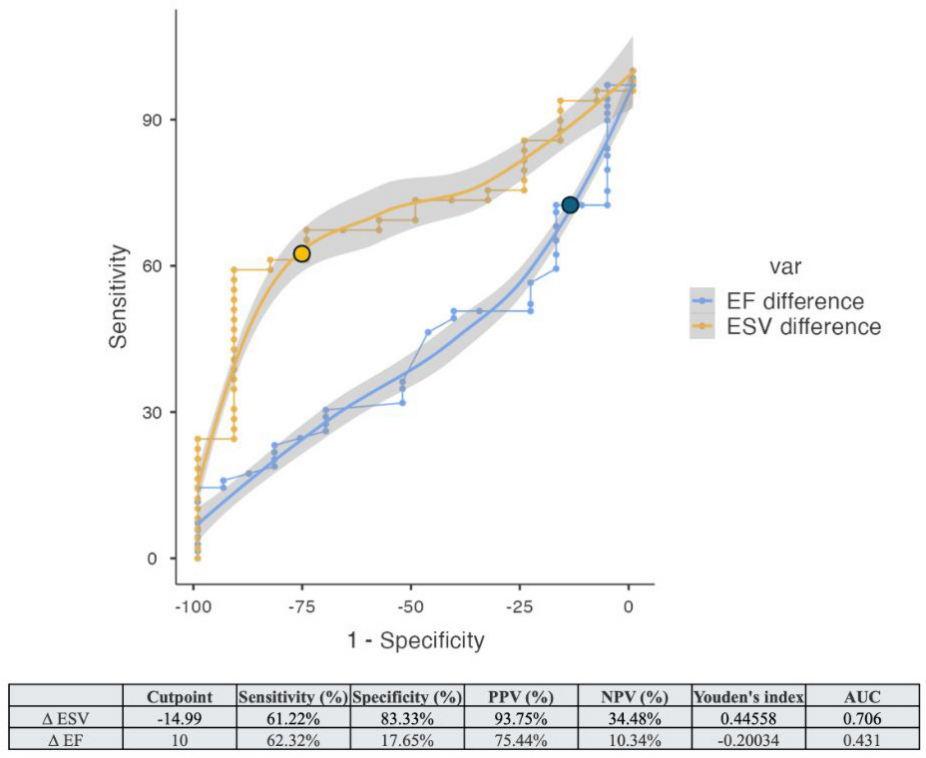
**ROC-curve analysis for LVEF difference and ESV difference to predict 2-year freedom from all cause mortality and hospitalization**. PPV, positive predictive value; NPV, negative Predictive value; ROC, receiver operating characteristic.

Univariate regression identified follow-up LVEF (OR 0.932; 95% CI: 0.881–0.986; *p* = 0.006), baseline RV ejection fraction (OR 0.958; 95% CI: 0.925–0.992; *p* = 0.015), baseline LV global longitudinal strain (OR 1.256; 95% CI: 1.003–1.57; *p* = 0.047), and baseline RV end-systolic volume index (OR 1.013; 95% CI: 1.000–1.025; *p* = 0.048) as significant predictors of events (shown in Table 3). ROC analysis showed that LVESV reduction ≥15% has a modest yet significant prediction for 2-year event-free survival (AUC: 0.706; sensitivity: 61.2%, specificity: 83.3%), whereas LVEF change lacked prognostic utility to predict the 2-year event-free survival (shown in Fig. 1).

**Table 3.** **Univariate predictors of all-cause mortality and hospitalization at two-year after CRT implantation**.

Variable		Odds ratio (OR)	95% confidence interval (CI)	*p*-value
Demographic/clinical variables:				
	Age at implant	0.974	0.943–1.01	0.11
	BMI	0.954	0.879–1.04	0.258
	6-minute walk test	1.001	0.994–1.01	0.723
Biomarkers:				
	NT-proBNP at baseline	1	1.000–1.000	0.484
	NT-proBNP at follow-up	1	1.000–1.000	0.134
	Creatinine at follow-up	2.353	0.925–5.988	0.072
	eGFR at baseline	0.988	0.974–1.00	0.105
	eGFR at follow-up	0.998	0.988–1.01	0.637
ECG parameters:				
	PR interval at baseline	1.012	0.982–1.04	0.434
	PR interval at follow-up	1.001	0.982–1.02	0.93
	QRS duration at baseline	1.003	0.974–1.03	0.842
	QRS duration at follow-up	1.007	0.980–1.03	0.612
	QTc interval at baseline	0.997	0.977–1.02	0.73
	QTc interval at follow-up	0.994	0.982–1.01	0.303
Device parameters:				
	Percent of biventricular pacing	1.06	0.819–1.37	0.658
Echocardiography:				
	LVEF at baseline	0.992	0.937–1.05	0.782
	LVEF at follow-up	0.932	0.881–0.986	0.006
	LVEDD at baseline	1.036	0.691–1.55	0.863
	LVESD at baseline	1.062	0.701–1.61	0.775
	LV ESV at baseline	1.001	0.996–1.01	0.631
	LVEDD at follow-up	1.15	0.811–1.63	0.432
	LVESD at follow-up	1.223	0.891–1.68	0.213
	LV ESV at follow-up	1.002	0.998–1.01	0.367
	LA Diameter	1.461	0.714–2.99	0.299
	E/A Ratio	1.455	0.896–2.36	0.13
	E/E′ Ratio	1.255	0.953–1.65	0.106
	TAPSE	0.909	0.793–1.04	0.168
	RV Diameter	0.857	0.590–1.24	0.417
	PASP	1.029	0.992–1.07	0.133
Cardiac MRI:				
	LVEF	0.965	0.915–1.02	0.177
	RVEF	0.958	0.925–0.992	0.015
	LVEDVi	0.999	0.992–1.01	0.783
	LVESVi	1	0.994–1.01	0.953
	LV Stroke Volume	0.982	0.962–1.00	0.101
	RVEDVi	1.007	0.995–1.02	0.241
	RVESVi	1.013	1.000–1.025	0.048
	RV stroke volume	0.98	0.957–1.00	0.081
	LV global longitudinal strain	1.256	1.003–1.57	0.047
	LV global circumferential strain	1.072	0.903–1.27	0.427
	LV global radial strain	0.942	0.864–1.03	0.178
	RV global longitudinal strain	1.102	0.965–1.26	0.153
	RV global circumferential strain	0.978	0.825–1.16	0.795

LVEDVi, left ventricular end diastolic volume indexed; LVESVi, left ventricular end-systolic volume indexed; RVEDVi, right ventricular end diastolic volume indexed; RVESVi, right ventricular end-systolic volume indexed.

### 3.4 Multivariate Model Performance

A multivariate model incorporating QRS shortening (≥20 ms), LVESV reduction (≥15%), and LVEF improvement (≥10%) was highly predictive of 2-year event-free survival (AUC 0.835, accuracy 84%, sensitivity 30%, specificity 97.5%; *p* = 0.006)., with QRS duration response ≥20 ms being the strongest independent predictor (adjusted OR 0.111, 95% CI 0.017–0.71; *p* = 0.02). Multivariable model for predicting All-cause mortality and hospitalization at two-year after CRT implantation is demonstrated in Table 4. The wide confidence intervals observed in multivariable analyses reflect the limited number of outcome events. Accordingly, these findings should be interpreted cautiously and viewed as hypothesis-generating, pending validation in larger cohorts.

**Table 4.** **Multivariable model for predicting all-cause mortality and hospitalization two-year after CRT implantation**.

Predictor	Adjusted OR	95% CI	*p*-value
Ejection fraction responders (≥10%)	0.38	0.065–2.27	0.29
End-systolic volume responders (≥15%)	0.175	0.027–1.13	0.067
QRS response (≥20 ms)	0.111	0.017–0.71	0.02

Model Performance: Area Under ROC Curve: 0.835, Accuracy: 84%, Sensitivity: 30%, Specificity: 97.5%. Note: Overall model *p*-value = 0.006. CRT, cardiac resynchronization therapy.

## 4. Discussion

This study investigated clinical, electrocardiographic, biomarker, echocardiographic, and CMR parameters to identify predictors of 2-year all-cause mortality and hospitalization following CRT implantation. Our analysis demonstrates that the synergistic integration of electrophysiological response (i.e., QRS narrowing) and structural/functional remodeling responses (i.e., LVEF improvement, LVESV reduction) provides superior predictive accuracy for long-term event-free survival compared to evaluating parameters in isolation. Several CRT response scores and composite endpoints have been proposed previously; however, most emphasize either clinical status or structural remodeling [10]. The present study adds incremental value by directly integrating electrical resynchronization with functional and structural remodeling within the same cohort and demonstrating that electrical response contributes prognostic information beyond reverse remodeling alone. Our findings suggest that CRT success is best conceptualized as a multidimensional physiological state rather than a single surrogate marker.

### 4.1 Baseline Prognostic Indicators

Patients experiencing clinical events within two years exhibited significantly poorer baseline prognostic markers compared to the event-free group. This included elevated NT-proBNP (*p* = 0.012) and serum creatinine (*p* = 0.028) levels, consistent with established literature linking higher natriuretic peptides [11] and impaired renal function [12] to adverse outcomes in CRT recipients. Furthermore, CMR assessment revealed significantly impaired right ventricular function in the event group, characterized by lower RVEF (*p* = 0.007) and higher RVESV (*p* = 0.005). While the prognostic significance of baseline RV function in CRT remains debated in the literature [13,14,15,16], our findings strongly support its predictive value. Baseline LV GLS also emerged as a significant predictor of events (*p* = 0.031), supporting prior evidence that abnormal pre-implant LV GLS portends worse long-term outcomes [17].

### 4.2 CRT Response Metrics as Outcome Predictors

Follow-up assessment at 6–12 months revealed marked differences in treatment response between groups. The event-free cohort demonstrated significantly greater improvements, including lower NT-proBNP levels (*p* = 0.01), higher LVEF (*p* = 0.008), and greater LVESV reduction (*p* = 0.029). Response rates further underscored this disparity: patients remaining event-free were significantly more likely to achieve a QRS response (≥20 ms narrowing; *p* = 0.006), LVEF response (≥10% absolute improvement; *p* < 0.001), LVESV response (≥15% reduction; *p* < 0.001), and BNP response (≥30% NT-proBNP decline; *p* = 0.024). This aligns with previous studies correlating positive outcomes with LVESV reduction [18], LVEF improvement [19], and BNP reduction [7,20] after CRT implantation. These findings strongly emphasize that achieving a positive physiological response to CRT is fundamental for long-term clinical benefit.

### 4.3 Predictors of All-Cause Mortality and Hospitalization After CRT

Univariate analysis identified key baseline and dynamic predictors of 2-year event-free survival. Impaired baseline RVEF (OR 0.958, 95% CI 0.925–0.992; *p* = 0.015) and worse LV GLS (OR 1.256, 95% CI 1.003–1.57; *p* = 0.047) were significant baseline risk factors, consistent with studies emphasizing RV dysfunction [21,22] and abnormal deformation mechanics [17,23] as predictors of adverse CRT outcomes. Notably, while baseline LVEF was similar between groups, the absence of LVEF improvement post-CRT was a powerful univariate predictor of events (OR 0.932; 95% CI: 0.881–0.986; *p* = 0.006). This reinforces that CRT’s benefit stems not from baseline status alone, but from achieving dynamic reverse remodeling [24,25]. On the other side, higher RVESVi at follow-up (OR 1.013, 95% CI 1.000–1.025, *p* = 0.048) was associated with increased risk of events, suggesting persistent RV maladaptation portends poor prognosis.

Crucially, our multivariate regression model demonstrated that a composite favorable response—defined by concurrent achievement of QRS narrowing ≥20 ms, LVESV reduction ≥15%, and LVEF improvement ≥10% at 6–12 months—was a highly significant predictor of event-free survival (Overall model *p* = 0.006). Although the composite response model demonstrated excellent specificity (97.5%) and discrimination (AUC 0.835), sensitivity was modest (30%). This indicates that while the model effectively identifies patients with a highly favorable prognosis, it is less effective at detecting all patients who will experience adverse events. Accordingly, the model should be viewed as a “rule in” prognostic tool rather than a screening instrument, particularly suitable for identifying patients with robust CRT benefit. QRS response ≥20 ms was the strongest independent predictor within the model (adjusted OR 0.111, 95% CI 0.027–1.13; *p* = 0.02), emphasizing that while electrical resynchronization is fundamental, integrating it with structural/functional remodeling metrics offers superior prognostic stratification [10,26]. This supports the findings of the previous studies where QRS narrowing was a major determinant of mortality [5,27], but contrasts with Lund-Andersen et al. [8], who found no mortality association with QRS shortening. This discrepancy may reflect differences in cohort characteristics, endpoint definitions (mortality vs. mortality/hospitalization), or, critically, the absence of combined remodeling assessment in their model [8]. Our data suggest QRS narrowing gains maximal prognostic significance when integrated with structural and functional response metrics.

### 4.4 Conduction System Pacing as an Emerging Alternative to CRT

In parallel with advances in CRT optimization, conduction system pacing (CSP), including His bundle pacing and left bundle branch area pacing, has emerged as a potential alternative strategy for electrical resynchronization. CSP aims to restore physiological ventricular activation by directly engaging the native conduction system and has shown promising effects on QRS narrowing and left ventricular function in selected patients [28]. However, contemporary evidence remains conflicting. A recent expert debate by Burri et al. [28] highlighted both the theoretical advantages and practical limitations of CSP compared with conventional biventricular pacing, including higher implantation complexity and variable long-term lead performance. A 2026 meta-analysis by Jin et al. [29] suggested improved electrical and echocardiographic responses with CSP in pacing-induced cardiomyopathy, though with substantial heterogeneity and limited randomized data. Conversely, Nielsen et al. [30] reported opposing results from two randomized trials comparing CSP and CRT, underscoring ongoing uncertainty regarding patient selection and durability of benefit.

In this context, our findings reinforce the central importance of achieving durable electrical resynchronization and reverse remodeling—regardless of pacing modality. Future comparative trials integrating multimodal response metrics, as proposed in our study, may help refine selection between CRT and CSP strategies.

## 5. Limitations

The small sample size and limited number of outcome events reduce statistical power and model robustness, as reflected by wide confidence intervals; therefore, the findings should be considered hypothesis-generating and require validation in larger cohorts. The relatively young, less comorbid population limits generalizability. Response assessment within a 6–12 month window introduced heterogeneity, and data on medical therapy optimization, cause-specific hospitalizations, and scar burden were limited. The observational design precludes causal inference, and the highly specific but low-sensitivity composite model should be interpreted as a post-implant risk stratification tool.

## 6. Conclusion

Our study reinforces the notion that a comprehensive assessment of CRT response, incorporating electrophysiological and cardiac remodeling parameters, is a powerful determinant of long-term event-free survival. Patients demonstrating combined improvements in QRS duration, LVESV, and LVEF after CRT implantation exhibit a significantly higher likelihood of 2-year event-free survival. While not designed as a screening tool, this pragmatic framework may complement existing CRT response definitions and support post-implant risk stratification.

## Data Availability

Data will be available upon reasonable request from the corresponding author.
